# Successful Non‐Coronary Cusp Ablation in Type B Wolff–Parkinson–White Syndrome With an rS Pattern in Lead V1

**DOI:** 10.1002/joa3.70527

**Published:** 2026-11-14

**Authors:** Yasuteru Yamauchi, Yuichiro Sagawa, Kazuya Murata, Atsuhito Oda, Kazutaka Aonuma

**Affiliations:** ^1^ Department of Cardiology Japan Red Cross Yokohama City Bay Hospital Yokohama Kanagawa Japan; ^2^ Department of Cardiology Mito Saiseikai General Hospital Mito Ibaraki Japan

**Keywords:** accessory pathway, catheter ablation, electrocardiography, noncoronary cusp, Wolff–Parkinson–White syndrome

## Abstract

A Type B Wolff–Parkinson–White pattern with an rS morphology in Lead V1 suggested a right anterior or anterolateral accessory pathway, whereas detailed activation mapping identified a para‐Hisian accessory pathway. Despite atypical ECG features, including relatively large initial r‐wave amplitudes in Leads V1 and V2, radiofrequency ablation from the noncoronary cusp successfully eliminated the pathway. This case highlights the limitations of ECG‐based localization and supports consideration of the noncoronary cusp when ablation from the right para‐Hisian region is unsuccessful.

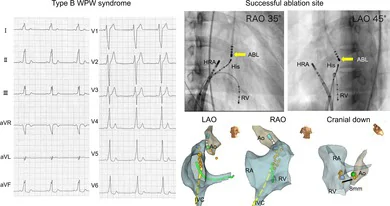

## 1 Introduction

Surface electrocardiographic localization of accessory pathways (APs) is important for planning catheter ablation. The Arruda algorithm is widely used to predict AP location based on initial delta‐wave polarity in multiple leads [1]. However, ECG‐based algorithms may be less reliable for APs with unusual anatomical courses, resulting in discrepancies between the predicted and actual AP locations. Para‐Hisian APs are particularly challenging because radiofrequency (RF) ablation near the His bundle carries a risk of atrioventricular (AV) conduction injury, whereas ablation from the noncoronary cusp (NCC) may provide an alternative approach [2, 3, 4]. We report a patient with Type B Wolff–Parkinson–White syndrome whose ECG suggested a right anterior or anterolateral AP, whereas mapping identified a para‐Hisian AP successfully eliminated by RF ablation from the NCC.

## 2 Case Report

A 20‐year‐old man with recurrent palpitations was referred to our institution after two previous ablation procedures that failed to eliminate the right‐sided accessory pathway. Five years earlier, manifest Wolff–Parkinson–White syndrome had been diagnosed, and a right anteroseptal AP had been ablated but rapidly recurred. During a second procedure, right anteroseptal and left lateral APs were identified. The left lateral AP was successfully eliminated, whereas the right‐sided AP could not be safely ablated because of its proximity to the His bundle.

The baseline ECG showed type B WPW syndrome with a distinct rS morphology in Lead V1 and positive initial delta‐wave components in Leads V1 and aVF. According to the Arruda algorithm, these findings suggested a right anterior or anterolateral AP (Figure 1A) [1].

**FIGURE 1:**
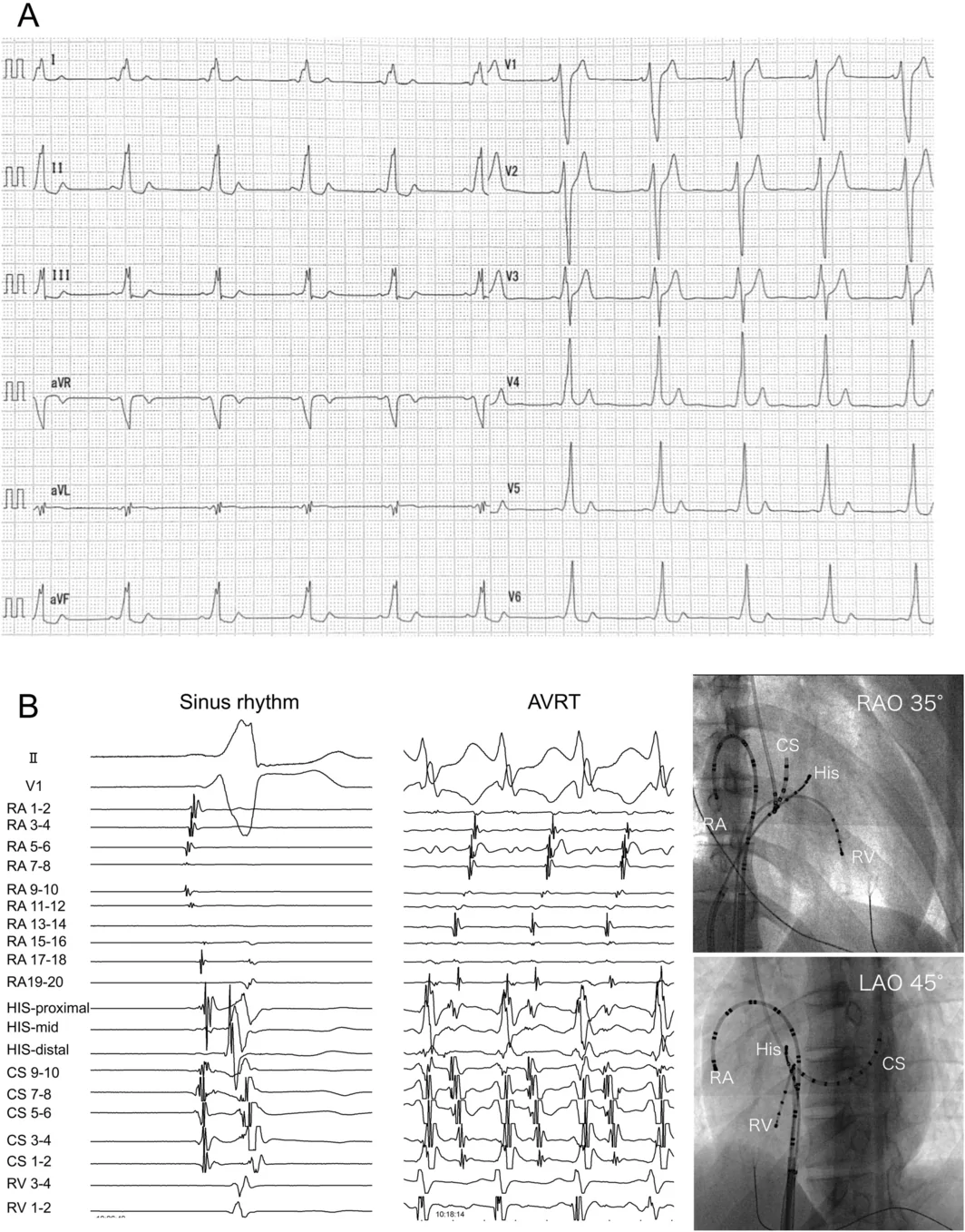
Surface electrocardiogram and baseline electrophysiological findings in type B Wolff–Parkinson‐White syndrome. (A) The delta waves were positive in Leads I, II, III, aVF, and V1. According to the Arruda algorithm, these findings suggested a right anterior or anterolateral accessory pathway. The initial r‐wave amplitudes in Leads V1 and V2 were 0.5 and 0.9 mV, respectively. (B) The earliest ventricular activation during sinus rhythm and the earliest atrial activation during orthodromic atrioventricular reentrant tachycardia (AVRT) were both localized to the distal His bundle region, suggesting a para‐Hisian accessory pathway. ABL, ablation catheter; CS, coronary sinus; HIS, His bundle; RA, right atrium; RV, right ventricle.

During the electrophysiological study, orthodromic AV reentrant tachycardia (AVRT) was reproducibly induced. Contrary to the ECG‐based prediction, the earliest ventricular activation during sinus rhythm was recorded at the distal His bundle region, suggesting a para‐Hisian AP. During ventricular pacing and AVRT, the earliest retrograde atrial activation was also recorded in the right anteroseptal region (Figure 1B). Using the EnSite mapping system, the earliest atrial activation during AVRT was precisely localized to the anteroseptal region at the His bundle recording site (Figure 2A). Similarly, during sinus rhythm, the earliest ventricular activation was identified on the right ventricular anterior wall adjacent to the His bundle recording site (Figure 2B).

**FIGURE 2:**
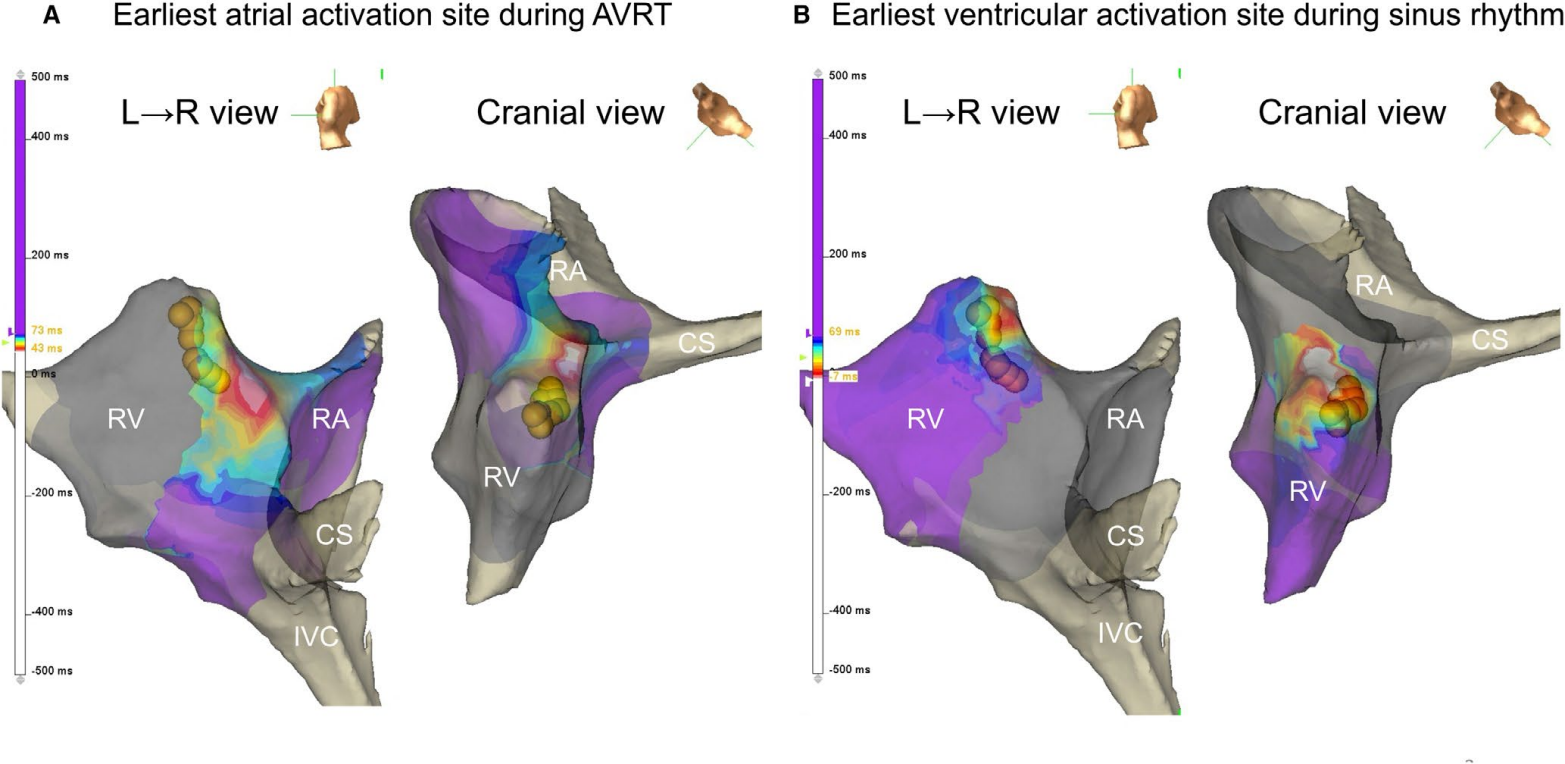
Electroanatomical mapping of retrograde and antegrade conduction through a para‐Hisian accessory pathway. (A) During AVRT, the earliest atrial activation was localized to the anteroseptal region at the His bundle recording site. (B) During sinus rhythm, the earliest ventricular activation was identified on the right ventricular anterior wall adjacent to the His bundle recording site. CS, coronary sinus; RA, right atrium; RV, right ventricle; IVC, inferior vena cava.

Because of its proximity to the normal conduction system, cryoablation was initially attempted from the right anteroseptal tricuspid annulus. At the earliest site, where the ventricular electrogram preceded the delta wave by approximately 30 ms, cryoablation resulted in only transient AP block. A subvalvular approach via the right jugular vein similarly demonstrated ventricular activation approximately 30 ms before the delta wave, but RF ablation was unsuccessful.

The aortic root was subsequently mapped using a transaortic approach. During AVRT, the local atrial activation time at the NCC was comparable to that recorded at the His bundle recording site (Figure 3A). RF energy was delivered with an irrigated‐tip catheter at 30 W for 60 s. AVRT terminated with ventriculoatrial block 5.5 s after RF initiation, followed by complete disappearance of the delta wave, confirming elimination of both retrograde and antegrade AP conduction (Figure 4). Three‐dimensional mapping demonstrated that the successful NCC ablation site was approximately 8 mm from the earliest right atrial activation site in the right para‐Hisian region (Figure 3B). No AV conduction disturbance or other complications occurred. The patient remained free of preexcitation and recurrent tachycardia during 3 years of follow‐up.

**FIGURE 3:**
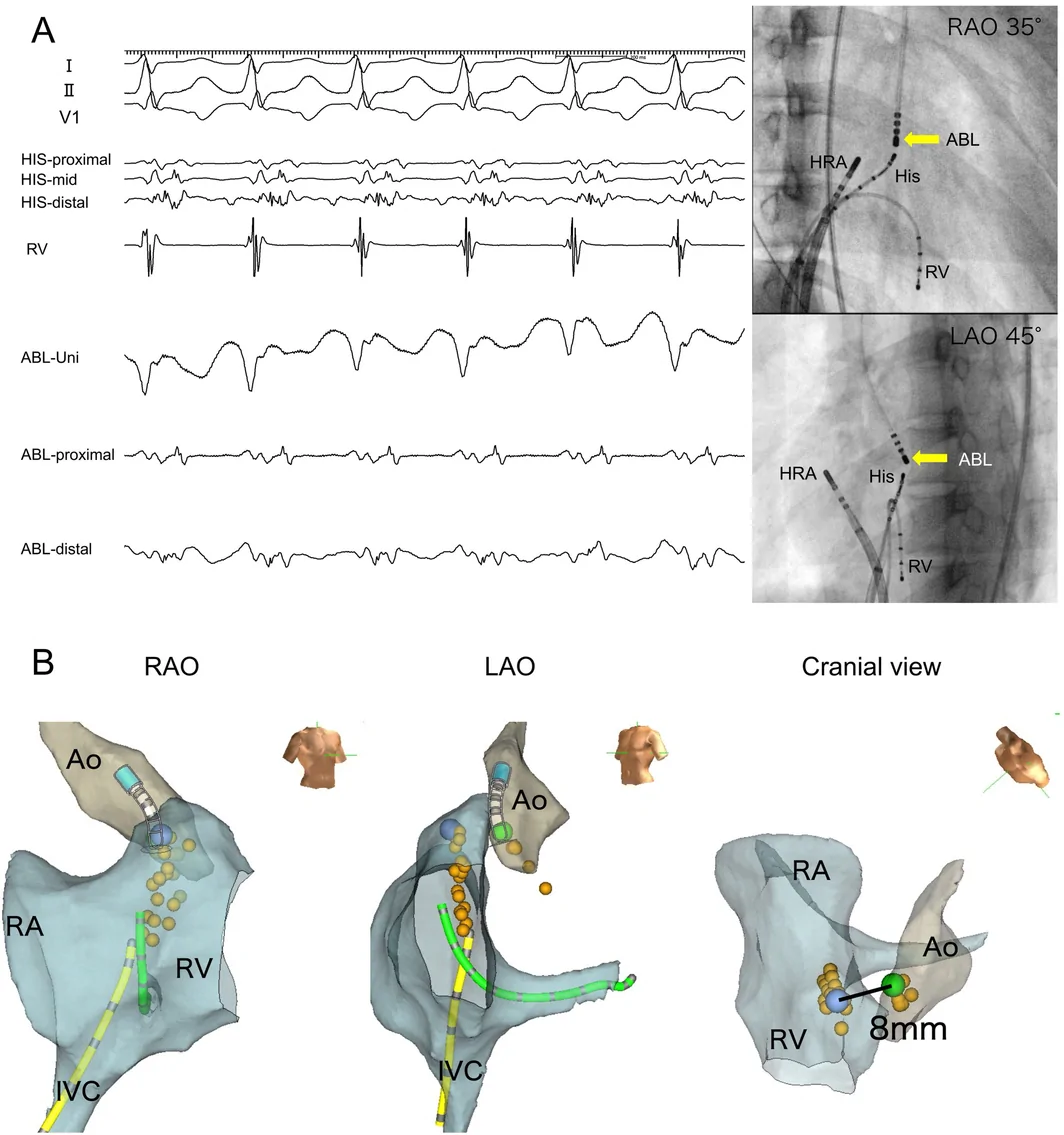
Intracardiac electrograms, catheter positions, and EnSite electroanatomical maps of the right atrium and aorta at the successful ablation site during orthodromic atrioventricular reentrant tachycardia. (A) The ablation catheter (yellow arrow) was positioned within the noncoronary cusp (NCC), where the local atrial electrogram showed activation timing comparable to that recorded by the His bundle catheter. (B) The green tag indicates the successful ablation site within the NCC, the yellow tags indicate sites where His bundle potentials were recorded, and the blue tag indicates the earliest atrial activation site in the right atrium. The earliest right atrial activation site and the successful NCC ablation site were located opposite each other, approximately 8 mm apart. ABL, ablation catheter; Ao, aorta; RA, right atrium; HIS, His bundle; HRA, high right atrium; IVC, inferior vena cava; LAO, left anterior oblique; RAO, right anterior oblique; RV, right ventricle.

**FIGURE 4:**
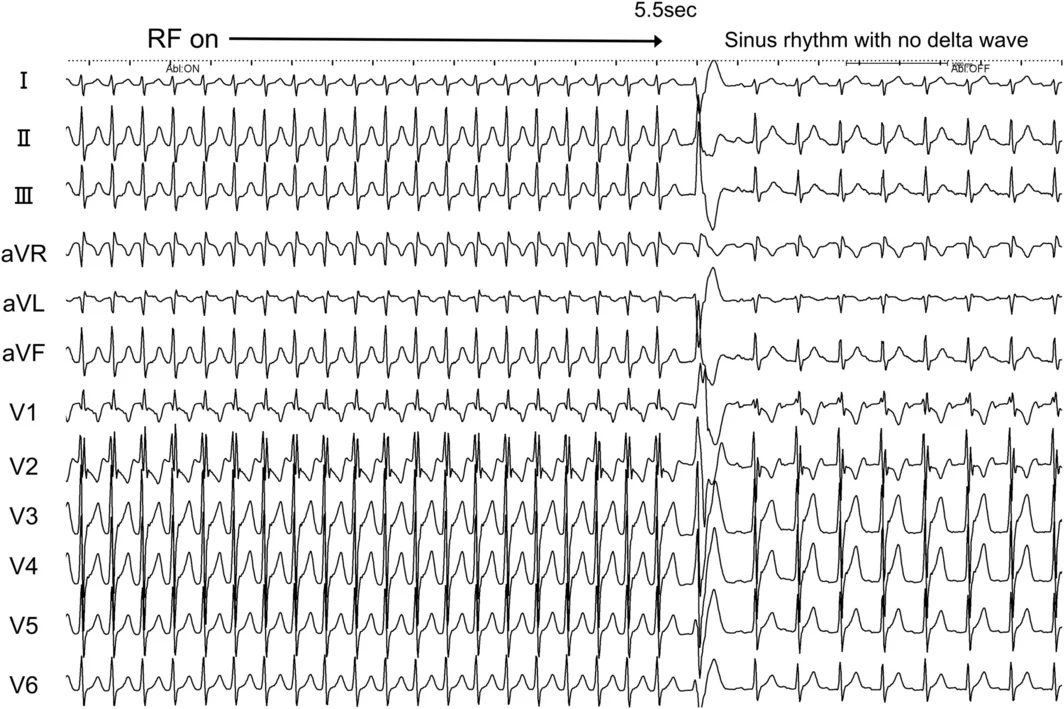
Termination of atrioventricular reentrant tachycardia during noncoronary cusp ablation. Radiofrequency (RF) energy was delivered at 30 W within the NCC. AVRT terminated with ventriculoatrial block 5.5 s after the initiation of RF delivery, followed by restoration of sinus rhythm with complete disappearance of the delta wave. No subsequent retrograde accessory pathway conduction was observed, confirming complete elimination of accessory pathway conduction.

## 3 Discussion

This case demonstrates a discrepancy between the AP location predicted from the surface ECG and that identified by electroanatomical mapping. Although the Arruda algorithm suggested a right anterior or anterolateral AP, [1] detailed mapping identified a para‐Hisian AP that was successfully eliminated from the NCC. The NCC is immediately adjacent to the interatrial septum and anteroseptal atrial myocardium. Myocardial extensions around the aortic root may form atrioventricular connections, providing an anatomical substrate for APs accessible from the NCC [2]. NCC ablation is an effective alternative for para‐Hisian APs when right‐sided ablation is unsuccessful or carries a risk of AV conduction injury [2, 3, 4]. Xu et al. reported a higher success rate and fewer complications with NCC ablation than with right anteroseptal ablation [3].

Importantly, APs accessible from the NCC may exhibit variable preexcitation patterns in the right precordial leads. Letsas et al. reviewed 36 septal APs successfully ablated from the aortic cusps, two‐thirds of which were ablated from the NCC [4]. Delta‐wave polarity in Lead V1 varied among APs ablated from the aortic cusps and was not site‐specific. Specifically, NCC APs could exhibit either positive or negative delta waves in lead V1 [4]. Thus, a positive initial deflection in Lead V1 does not exclude a para‐Hisian AP accessible from the NCC.

González‐Torrecilla et al. investigated ECG features distinguishing strict para‐Hisian APs from anteroseptal and midseptal APs [5]. Among 52 strict para‐Hisian APs, the sum of the initial r‐wave amplitudes in Leads V1 and V2 was significantly smaller than that in the control group, which predominantly comprised anteroseptal and midseptal APs (0.26 ± 0.22 vs. 0.71 ± 0.36 mV) [5]. A cutoff of < 0.5 mV identified a strict para‐Hisian location with 85% sensitivity and 75.5% specificity [5]. Notably, the individual patient data reported by González‐Torrecilla et al. showed that the sum of the initial r‐wave amplitudes in Leads V1 and V2 was < 1.0 mV in all 52 para‐Hisian APs [5]. In contrast, the corresponding value in our patient was 1.4 mV. Thus, the prominent initial positive deflections in Leads V1 and V2 were atypical even according to a quantitative criterion specifically developed for strict para‐Hisian APs. This discrepancy may reflect differences in AP anatomy. Importantly, APs located at the NCC were not represented in the González‐Torrecilla series [5]. Their ECG criteria therefore may not apply to para‐Hisian APs accessible from the NCC. Although Letsas et al. demonstrated substantial heterogeneity in Lead V1 delta‐wave polarity among septal APs successfully ablated from the aortic cusps, they did not assess the initial r‐wave amplitudes in leads V1 and V2 [4]. These findings suggest that para‐Hisian APs accessible from the NCC may exhibit ECG characteristics that differ from those of para‐Hisian APs amenable to ablation from the right atrial septum. Further studies are needed to determine whether distinct ECG characteristics can differentiate these two groups. Nevertheless, when an AP is identified as para‐Hisian by right atrial mapping but cannot be successfully eliminated by ablation from the right atrial septum or a subvalvular approach, the NCC should be considered as an alternative mapping and ablation site, even when the ECG shows a positive delta wave in Lead V1 and relatively large initial r‐wave amplitudes in leads V1 and V2, suggesting a right anterior AP.

## Funding

The authors have nothing to report.

## Ethics Statement

This case report was approved by the Japan Red Cross Yokohama City Bay Hospital Ethics Committee.

## Consent

Written informed consent was obtained from the patient.

## Conflicts of Interest

The authors declare no conflicts of interest.

## Data Availability

The data that support the findings of this study are available from the corresponding author upon reasonable request.
